# Incidence of cardiometabolic diseases in a Lesotho HIV cohort: Evidence for policy decision-making

**DOI:** 10.4102/sajhivmed.v22i1.1246

**Published:** 2021-06-28

**Authors:** Motlalepula Sebilo, Neo R.T. Ledibane, Simbarashe Takuva

**Affiliations:** 1 School of Health Systems and Public Health, Faculty of Health Sciences, University of Pretoria, Pretoria, South Africa; 2 Elizabeth Glaser Pediatric AIDS Foundation, Maseru, Lesotho; 3 Perinatal HIV Research Unit, Faculty of Health Sciences, University of the Witwatersrand, Johannesburg, South Africa

**Keywords:** AIDS, antiretroviral treatment, cardiometabolic disease, HIV, incidence rate, Lesotho

## Abstract

**Background:**

Antiretroviral treatment (ART) has been associated with the development of certain cardiometabolic diseases (CMDs). The burden of CMDs amongst ART-experienced patients in sub-Saharan Africa was unknown.

**Objective:**

We quantified the burden of CMDs and identified the associated risk factors in a large treatment cohort on ART at a high-volume facility in Lesotho.

**Methods:**

In this retrospective cohort study, we extracted data from the daily dispensing electronic system and routine clinical records of 785 adults on ART between 2011 and 2015 in Maseru, Lesotho. CMD was defined as a diagnosis of hypertension, diabetes mellitus or dyslipidaemia (singly or collectively). Descriptive statistics were used to describe the disease burden; Kaplan–Meier curves and cause-specific Cox proportional hazards models were fitted to examine the impact of the ART regimen and identify the risk factors associated with the occurrence of CMD.

**Results:**

Of the 785 participants, 473 (60%) were women. The median age of the group was 42 years, interquartile range (IQR), 36–51 years. The overall incidence of CMD was 5.6 (95% confidence interval [CI] = 4.4–7.1) per 100 person-months of follow-up. The median time to onset of CMD was 16.6 months (IQR = 7.4–23.4). ART was not associated with the occurrence of CMD (cause-specific hazard ratio [CHR] = 1.55; 95% CI = 0.14–16.85; *P* = 0.72). Higher body mass index (BMI) was associated with the occurrence of diabetes mellitus (CHR = 1.19; 95% CI = 1.14–1.38; *P* = 0.026).

**Conclusion:**

The incidence of CMD in this relatively young patient population is low yet noteworthy. We recommend that patients living with HIV and AIDS should be routinely screened for CMD. Higher BMI is generally associated with the occurrence of CMD.

## Introduction

Sub-Saharan Africa (SSA) currently has the largest burden of HIV infection in the world and 69.65% of people living with HIV (PLWH) were reported to be on antiretroviral treatment (ART) in 2019.^1^ The countries with the highest burden of HIV include Swaziland, Botswana and Lesotho, with approximately 25% of the general population infected.^2^ The uptake of ART has been increasing annually with the scaling up of ART services in response to the Joint United Nations Programme on HIV/AIDS (UNAIDS) 95-95-95 targets, where 95% PLWH know their status, 95% of people who know their status are accessing treatment and 95% of people on treatment have suppressed viral loads.^3^

Since the introduction of ART, the survival and life expectancy of PLWH have been enhanced, with approximately 19.1 million life-years being saved from 1990 to 2013.^4,5^

Prolonged exposure to ART, in addition to aging because of improved life expectancy, is associated with a higher risk of cardiometabolic diseases (CMDs), which include dyslipidaemia, obesity, hyperglycaemia (diabetes mellitus) and hypertension. Antiretroviral treatments, specifically protease inhibitors (PIs), are associated with the accelerated progression of metabolic complications in PLWH, while some non-nucleoside reverse transcriptase inhibitors (NNRTIs) are also reported to have metabolic side-effects.^6,7^

Since the introduction of ART, approximately 4 million PLWH have survived beyond 50 years of age.^8^

Despite good management and control, elderly patients on ART have a higher risk of CMDs (which tends to appear approximately 10 years earlier than in HIV-uninfected individuals) compared to their counterparts in the general population, because of interactions between the natural risk factors that develop with aging and the effects of ART and HIV.

Cumulative exposure to ART is associated with an increase in the risk of myocardial infarction per year of exposure (relative risk = 1.12; 95% confidence interval [CI] = 1.06–1.18).^9^ Exposure to ART for longer than 6 months is also associated with higher serum triglycerides levels, total cholesterol, low-density lipoprotein cholesterol, insulin resistance and cardiovascular disease.^10,11,12^

The global estimated prevalence of hypertension is 34.7% among ART-experienced patients and 12.7% among ART-naïve patients.^13^ There is a dearth of accurate estimates for the global prevalence and incidence of diabetes mellitus and dyslipidaemia among ART-experienced patients. In Brazil, patients who had been on ART for three years had a cumulative incidence of dyslipidaemia of 22.3% and diabetes mellitus of 5.8%.^14^

The burden of CMD in SSA is increasing parallel to the changing landscape of HIV care. It is projected that deaths attributed to cardiovascular disease will double to 2.4 million in 2030 relative to reports from 2000.

These data suggest that cardiovascular disease is becoming a major public health problem in SSA, competing with infectious diseases for limited health resources.^15^

Generally, little is known about ART-associated CMDs among ART-experienced PLWH in SSA, despite the region having the greatest burden of HIV infection and increasing access to ART.^16,17^ The understanding of the effect of ART on the risk of CMDs is crucial to enable an accurate estimation and the assessment of appropriate interventions for the management of CMDs in PLWH.^18,19^ Understanding the interaction between ART and CMD is crucial for managing CMDs in PLWH.^18,19^ Being part of SSA, Lesotho is no exception and needs to be studied.

There were approximately 306 000 PWLH in 2016/2017 in Lesotho, corresponding to 25.6% of the population.^20,21^ Of these, approximately 70% were enrolled in ART care and treatment.^20,21^ Lesotho thus has one of the highest burdens of HIV globally. In this study, we quantified the burden of CMD and identified the associated risk factors among PLWH in a large treatment cohort.

## Methods

### Study design and setting

This retrospective cohort study used data from the daily dispensing electronic system and routine clinical records at Qoaling Filter Clinic, Maseru, South Africa, for the period May 2011 to May 2015.

Qoaling Filter Clinic is a high-volume public–private partnership primary healthcare facility located in Qoaling constituency, which is approximately 8 km from the capital city of Lesotho, Maseru. In addition to HIV and AIDS care and tuberculosis (TB) management services, the clinic offers dental services, ophthalmology, antenatal care, pharmacy services, family planning, minor procedures and wound care services and outpatient department services for minor ailments and some chronic conditions. Patients access services for a fee subsidised by the government of Lesotho except for HIV and AIDS care services, which are free of charge.

In Lesotho, ART is currently initiated in all people testing positive for HIV as soon as they are ready to commit to treatment, regardless of the availability of baseline laboratory tests.

However, prior to 2016, a thorough clinical evaluation coupled with baseline laboratory investigations was mandatory to determine the patients’ eligibility for ART. Adults and adolescents were eligible for ART initiation under certain conditions, that is if they were at World Health Organization (WHO) clinical stage 3 or 4 (regardless of CD4 count) or WHO clinical stage 1 or 2 (CD4 ≤ 500 cells/mm^3^), HIV serodiscordant couples with TB or hepatitis B co-infection, and HIV-infected women who were pregnant or lactating. Prior to ART initiation, mandatory baseline laboratory investigations are CD4 count, cryptococcal antigen screening for adolescents and adults with CD4 counts of < 100 cells/mm, hepatitis C serology, haemoglobin (Hb) or full blood count, Alanine Aminotranferase (ALT) and creatinine, urine dipstick (glucose, protein), pregnancy test, blood glucose, cholesterol and triglycerides for PI-based regimens.^22^ The country’s 2014 guidelines on the use of ART for HIV prevention and treatment recommended ART regimens are shown in Table 1.^22^

**TABLE 1:** Recommended antiretroviral treatment regimens for adolescents and adults in Lesotho.

First line	Second line	Third line
Preferred first-line regimen: Tenofovir/lamivudine/efavirenz	Tenofovir/lamivudine/(lopinavir/ritonavir) or (atazanavir/ritonavir) Zidovudine/lamivudine/(lopinavir/ritonavir) or (atazanavir/ritonavir)	Third-line drugs for Lesotho are as follows: darunavir, ritonavir as a pharmacokinetic booster, raltegravir and etravirine.
Alternatives: Tenofovir/lamivudine/nevirapine Zidovudine/lamivudine/efavirenz Zidovudine/lamivudine/nevirapine Abacavir/lamivudine/efavirenz Abacavir/lamivudine/nevirapine	Abacavir/lamivudine/(lopinavir/ritonavir) or atazanavir/ritonavir	-

Source: MOH. National guidelines on the use of ART for HIV prevention and treatment [homepage on the Internet]. 2014 [cited 2020 Oct 17]. Available from: https://hivstar.lshtm.ac.uk/files/2017/11/NATIONAL-GUIDELINES-ON-THE-USE-OF-ART-FOR-HIVPREVENTION-AND-TREATMENT-4TH-ED_lesotho_2014.pdf

Because of its lower potency and increased risk of liver toxicity when initiated at CD4 counts of ≥ 250 cells/mm^3^ in women and ≥ 400 cells/mm^3^ in men, nevirapine is no longer recommended as part of first-line ART regimens. However, HIV-infected patients who are already on a nevirapine-based regimen and stable should continue using the regimen and multidrug-resistant TB (MDR-TB) co-infected patients already on nevirapine because of interactions between MDR-TB medication and efavirenz.

### Study population

Patient records were included in the study if the patient was older than 18 years old, initiated onto the standard first- or second-line ART regimen for one year or longer and had documented viral load measurements. Patients who already had diabetes mellitus, hypertension, obesity or dyslipidaemia at ART initiation were excluded.

### Sample size and sampling techniques

We calculated the required sample size as specified by a prospective cohort study of the incidence of lipodystrophy and metabolic disorders in patients starting on NNRTIs in Benin.^23^ Using STATA version 14 (Stata Corp., College Station, TX, United States [US]), we derived a minimum sample size of 279 participants – 118 men (hazard ratio [HR] = 6.33, standard deviation [s.d.] = 0.14, alpha = 0.05 and power = 0.80) and 161 women (HR = 6.33, s.d. = 0.12, alpha = 0.05 and power = 0.80). Given the available database of patients, we increased the detection value to an HR value of 3 or greater to achieve a more optimal minimum sample of 785 patients. After the above calculation, from the facility spreadsheet of approximately 4000 patients currently on ART, we filtered the data for eligible patients.

We then selected 785 patients through Microsoft Excel random selection function, and extracted the pertinent data from clinical records and the daily electronic dispensing system.

## Measurements

### Definition of the outcome

The primary outcome was the occurrence of CMD which include conditions like hypertension, diabetes mellitus or dyslipidaemia. We assumed that patients had hypertension if they were on antihypertensive medication, similarly had dyslipidaemia if they were on lipid-lowering medication and had diabetes mellitus if they were being treated for diabetes mellitus.

### Independent variables

The predictor variables included demographic and clinical characteristics such as age, gender, body mass index (BMI), viral load, CD4 cell count and a history of hypertension, diabetes mellitus or dyslipidaemia. The ART regimen was the main exposure variable. Other variables included treatment adherence, the date of ART initiation, ART, duration of ART in months, date of diagnosis and initiation dates of hypertension treatment, diabetes mellitus treatment and dyslipidaemia treatment.

### Data management and analysis

Data were captured using EpiData version 3.1 and verified using the double-entry method. Data were exported to STATA version 14.0 (Stata Corp.) for analysis.

The data were summarised using descriptive statistics. The mean and standard deviation were calculated for normally distributed numerical variables, while the median and interquartile ranges (IQRs) were used to describe the skewed numerical variables. The categorical variables were summarised using frequencies and proportions.

The person-time accrued was calculated from six months after treatment initiation, which is the earliest time possible for viral load monitoring and other recommended routine laboratory tests after baseline tests,^24^ until the earliest of (1) the study outcome (any one of the three CMDs); (2) death; (3) loss to follow-up (defined as at least 90 days late for the last scheduled clinic visit); (4) transferring out or (5) the close of the dataset (May 2015).

We measured the incidence of CMDs as the total number of new cases of CMD at every 6-month follow-up divided by the total number of patients at risk during the same period, expressed per 100 person-months of follow-up. The incidence rates were stratified by first-line and second-line regimens. To estimate the hazards of developing CMD beyond a specified 6-month period, we generated Kaplan–Meier survival curves. The log-rank test was used to compare the survival curves of participants on NNRTI and PI-based regimens.^25^

All *P*-values < 0.05 were considered statistically significant. We used a cause-specific Cox proportional hazards regression model to investigate the impact of exposure to ART, as well as the effect of age, BMI, Hb, CD4 cell count and viral load on the development of CMDs.^26^

### Ethical considerations

This study was approved by the Ministry of Health (Lesotho) Research Ethics Committee (reference number 56/2019) and the University of Pretoria Faculty of Health Sciences Research Ethics Committee (reference number ID36-2019).

## Results

### Demographic and clinical characteristics of the study participants

For the period 2011–2015, we randomly selected 785 participants through the Microsoft Excel random selection (simple random sampling) programme. Most of the participants were women (60%, *n* = 473), with a median age of 42 years (IQR = 36–51) and a median duration on ART of 15 months (IQR = 3–49). The participants had a mean BMI of 25.4 kg/m^2^ (s.d. = 8.42) and a Hb level of 13.9 g/dL (s.d. = 1.82). Overall, 63.7% (*n* = 500) of participants had a CD4 count of < 500 copies/mm^3^. The participants were stratified according to their current ART regimen (NNRTI-based regimen: 98.1%, *n* = 770; PI-based regimen: 1.9%, *n* = 15). All the PI-based regimen participants were initiated onto second-line regimens and the clinical records revealed no history of the conditions under investigation at the beginning of the study. Description of the average demographic and clinical characteristics is given in Table 2.

**TABLE 2:** Description of the demographic and clinical characteristics of the study participants by antiretroviral treatment regimen.

Characteristic	NNRTI-based regimen (*N* = 770)						PI-based regimen (*N* = 15)						Total (*N* = 785)					
	*n*	%	Median	IQR	Mean	s.d.	*n*	%	Median	IQR	Mean	s.d.	*n*	%	Median	IQR	Mean	s.d.
**Age (years)**	-	-	42	36–51	-	-	-	-	43	37 – 55	-	-	-	-	42	36 – 51	-	-
**Gender**	-	-	-	-	-	-	-	-	-	-	-	-	-	-	-	-	-	-
Male	307	39.9	-	-	-	-	5	33.3	-	-	-	-	312	39.7	-	-	-	-
Female	463	60.1	-	-	-	-	10	66.7	-	-	-	-	473	60.3	-	-	-	-
**Body mass index (kg/m^2^)**	-	-	-	-	25.4	8.42	-	-	-	-	24.8	3.84	-	-	-	-	25.4	8.36
≤ 25kg/m^2^	445	57.8	-	-	-	-	7	46.7	-	-	-	-	452	57.6	-	-	-	-
˃ 25kg/m^2^	325	42.2	-	-	-	-	8	53.3	-	-	-	-	333	42.4	-	-	-	-
**Haemoglobin (g/dL)**	-	-	-	-	13.9	1.82	-	-	-	-	11.9	1.95	-	-	-	-	13.8	1.84
**Adherence (%)**	-	-	97.5	95–99	-	-	-	-	87.0	82.5–93	-	-	-	-	97.5	95.0–99.0	-	-
˂ 95%	204	26.5	-	-	-	-	13	86.7	-	-	-	-	217	27.6	-	-	-	-
≥ 95%	566	73.5	-	-	-	-	2	13.3	-	-	-	-	568	72.4	-	-	-	-
**Viral load**	-	-	31	20–10 875	-	-	-	-	10 875	4473–28 579	-	-	-	-	32.5	20.0–447.8	-	-
˂ 1000 copies/mL	705	91.5	-	-	-	-	2	13.3	-	-	-	-	707	90.1	-	-	-	-
≥ 1000 copies/mL	65	0.08	-	-	-	-	13	86.7	-	-	-	-	78	0.1	-	-	-	-
**CD4 count**	-	-	426	308–585	-	-	-	-	205	146–271	-	-	-	-	421	298–580	-	-
˂ 500 cells/mm^3^	486	63.1	-	-	-	-	14	93.3	-	-	-	-	500	63.7	-	-	-	-
≥ 500 cells/mm^3^	284	36.9	-	-	-	-	1	6.7	-	-	-	-	285	36.3	-	-	-	-

PI, protease inhibitor; NNRTI, non-nucleoside reverse transcriptase inhibitor; IQR, interquartile range; s.d., standard deviation.

### Incidence of cardiometabolic disease

Because there were only 15 second-line patients, in the interest of sample homogeneity, the main analysis of the study focused only on first-line patients. Additionally, there were only three cases of dyslipidaemia during the period of the study; therefore, they were also left out in the main analysis. Twelve (12) participants developed both hypertension and diabetes mellitus and there was only one case in each of the other multimorbidity combinations. The incidence rate was 5.6 (95% CI = 4.4–7.1), 5.7 (95% CI = 4.4–7.3) and 5.5 (95% CI = 3.3–8.9) per 100 person-months of follow-up for CMD, hypertension and diabetes mellitus, respectively (Table 3).

**TABLE 3:** Incidence rates for hypertension and diabetes mellitus among study participants who had been on antiretroviral treatment for more than 1 year in Lesotho (2011–2015).

Outcome	Person-time (months)	No. of cases	Incidence rate (per 100 person-months of follow-up)	95% CI
CMD	39.1	78	5.5	4.3–7.0
Hypertension	35.9	62	5.7	4.4–7.3
Diabetes mellitus	9.6	16	5.5	3.3–8.9

CMD, cardiometabolic disease; CI, confidence interval.

### Occurrence of cardiometabolic disease

**FIGURE 1:**
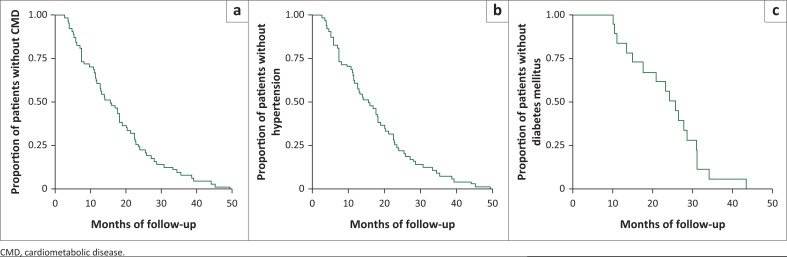
Kaplan–Meier time estimates to occurrence of cardiometabolic disease (a), hypertension (b) and diabetes mellitus (c).

Figure 1 shows the Kaplan–Meier survival curves for CMD, hypertension and diabetes mellitus outcomes. Patients’ clinical assessments are normally performed at 2 weeks, 1 month, 2 months, 3 months and 6 months after ART initiation and at least every 6 months thereafter complemented by laboratory monitoring. The viral load and CD4 count are routinely measured at 6 months and 12 months after ART initiation and annually thereafter;^22^ therefore, we found it relevant to use 6-month segments in our Kaplan–Meier survival curves. From the date of ART initiation, the median time to onset was 16.6 months for CMD (IQR = 7.4–23.4), 15.6 months for hypertension (IQR = 7.4–23.4) and 19.5 months for diabetes mellitus (IQR = 12.1–24.9).

### Factors associated with occurrence of either hypertension or diabetes mellitus

Antiretroviral treatment was not associated with the occurrence of any of the three distinct outcomes, CMD (cause-specific hazard ratio [CHR] = 1.55; 95% CI = 0.1–16.85; *P* = 0.158), hypertension (CHR = 2.10; 95% CI = 0.18–24.24; *P* = 0.552) or diabetes mellitus (CHR = 0.60; 95% CI = 0.09–1.83; *P* = 0.481). A higher BMI was significantly associated with the occurrence of diabetes mellitus (CHR = 1.19; 95% CI = 1.14–1.38; *P* = 0.026) (Table 4).

**TABLE 4:** Risk factors for cardiometabolic disease, hypertension and diabetes mellitus.

Variable	Unadjusted HR	95% CI	*P*-value	Cox PH model			Cause-specific Cox PH model		
				AHR	95% CI	*P*-value	CHR	95% CI	*P*-value
**Cardiometabolic disease outcome**									
Viral load (˂ 1000 vs ≥ 1000 copies/mL)	0.88	0.29–2.70	0.82	1.08	0.43–2.68	0.87	1.16	0.37–3.60	0.80
CD4 count (˂ 500 vs ≥ 500 cells/mm^3^)	1.37	0.77–2.44	0.29	1.36	0.77–2.40	0.29	1.38	0.73–2.62	0.32
ART	3.18	0.64–15.72	0.16	3.07	0.65–14.57	0.16	1.55	0.14–16.85	0.72
Age	1.04	0.99–1.08	0.12	1.03	0.99–1.08	0.14	1.01	0.96–1.05	0.93
BMI	1.06	1.11–1.12	0.05	1.06	1.01–1.11	0.02	1.05	0.98–1.11	0.03
Hb	1.19	0.98–1.43	0.07	1.18	0.00–1.40	0.067	1.03	0.85–1.24	0.78
Gender (female vs male)	0.98	0.47–2.08	0.97	-	-	-	-	-	-
Adherence (˂ 95 vs ≥ 95%)	0.79	0.37–1.69	0.50	-	-	-	-	-	-
**Hypertension outcome**									
Viral load (˂ 1000 vs ≥ 1000 copies/mL)	1.08	0.36–3.25	0.90	1.26	0.52–3.07	0.61	1.09	0.35–3.42	0.86
CD4 count (˂ 500 vs ≥ 500 cells/mm^3^)	1.41	0.78–2.56	0.25	1.37	0.76–2.46	0.30	1.41	0.74–2.67	0.80
ART	1.56	0.15–16.57	0.71	1.84	0.18–18.61	0.61	2.10	0.18–24.24	0.55
Age	1.02	0.97–1.06	0.38	1.02	0.98–1.06	0.38	1.01	0.97–1.06	0.57
BMI	1.06	1.00–1.12	0.03	1.06	1.00–1.10	0.07	1.02	0.96–1.08	0.47
Hb	1.14	0.96–1.36	0.14	1.15	0.98–1.38	0.09	1.01	0.84–1.22	0.86
Gender (female vs male)	1.47	0.71–3.04	0.30	-	-	-	-	-	-
Adherence (˂ 95 vs ≥ 95%)	0.86	0.39–1.93	0.73	-	-	-	-	-	-
**Diabetes mellitus outcome**									
Viral load (˂ 1000 vs ≥ 1000 copies/mL)	0.03	0.00–2.01	0.10	0.13	0.02–0.93	0.04	0.18	0.11–1.24	0.05
CD4 count (˂ 500 vs ≥ 500 cells/mm^3^)	0.31	0.03–3.65	0.35	0.51	0.14–1.91	0.32	0.58	0.19–1.21	0.33
ART	0.55	0.05–5.87	0.62	0.46	0.05–1.95	0.48	0.60	0.09–1.83	0.48
Age	1.02	0.86–1.20	0.84	0.99	0.85–1.14	0.32	1.03	0.88–1.46	0.33
BMI	1.15	0.98–1.35	0.09	1.16	1.02–1.33	0.02	1.19	1.14–1.38	0.03
Hb	1.54	0.82–2.88	0.18	1.34	0.83–2.17	0.13	1.46	0.87–2.19	0.14
Gender (female vs male)	0.53	0.02–12.99	0.70	-	-	-	-	-	-
Adherence (˂ 95 vs ≥ 95%)	0.23	0.01–6.27	0.38	-	-	-	-	-	-

HR, hazard ratio; AHR, adjusted hazard ratio; CHR, cause-specific hazard ratio; PH, proportional hazard; ART, antiretroviral treatment; BMI, body mass index; Hb, haemoglobin; CI, confidence interval.

## Discussion

We explored the incidence of and identified the risk factors for CMDs among PLWH in a large treatment cohort in Lesotho. In this relatively young cohort of PLWH, there was a low incidence of CMDs, with the treatment regimen having little effect on the incidence or the time to onset of CMDs. A higher BMI was the only predictive factor for diabetes mellitus. Our findings provide valuable baseline information about the influence of ART on the incidence of CMDs in PLWH in Africa, especially Lesotho, which has the second-highest prevalence of HIV after Eswatini.^2,27,28,29^

Because we randomly selected 785 patients from a treatment database, we were able to select a sample that was representative of the population available in our patient database. Our study comprised 60.3% (473) women and 39.7% (312) men, equivalent to the ratios reported in the 2019 UNAIDS report. The UNAIDS data indicated that the numbers of women and men older than 15 years living with HIV in 2018 were 190 000 and 130 000, a ratio of 59.4% women and 40.6% men, respectively.^30^ In our study, approximately 90% of patients were virally suppressed, which is similar to 88% reported in 2017.^31^ Most patients had a CD4 count below 500 cells/mm^3^, which is contrary to the main goals of ART. Antiretroviral treatment aims to achieve viral suppression in the shortest time possible, sustaining viral suppression and thus allowing patients’ CD4 counts to increase.^32^ Most patients in our study (72.4%) demonstrated good treatment adherence, taking at least 95% of the medication in the correct amount, at the correct time.^24^

### Incidence of cardiometabolic diseases

In Lesotho, PLWH had an incidence rate of hypertension of 5.7 (95% CI = 4.4–7.2) per 100 person-months of follow-up or a cumulative incidence of 8.5%, which is much lower than 34.7% reported by Xu et al.^13^

Prioreschi et al. reported incidence rates of type 2 diabetes mellitus amongst PLWH ranging from 4 to 59 per 1000 person-years in PLWH on ART.^33^

In our study, PWLH had a diabetes mellitus incidence rate within the above range: 5.3 (95% CI = 3.3–8.4) per 100 person-months of follow-up or 22.3 per 1000 person-years of follow-up.

The proportions and incidence rates reported in our study are lower than those reported in other studies; however, the future projections for non-communicable disease (NCD) burdens are concerning. It is projected that the proportion of people suffering from at least one key NCD in 2035 will increase by 26% among PLWH compared to 6% among people not living with HIV. Adult PLWH will be twice as likely to suffer from at least one key NCD in 2035 compared to uninfected adults.^34^

### Occurrence of cardiometabolic diseases

In our study, hypertension first occurred at approximately 6 months, which is similar to findings reported by Wu et al.^10^ As portrayed in Figure 1, gradual failure was observed for all outcomes although a little bit delayed to start on diabetes mellitus outcome.

### Factors associated with occurrence of either hypertension or diabetes mellitus

The main factors associated with metabolic disorders in PLWH include a high proportion of central body fat, older age, higher BMI and high calorie, sugar and carbohydrate intake.

The HIV-related factors include a high viral load and exposure to certain antiretroviral drugs, such as PIs.^35^

In this study, a higher BMI was significantly associated with the occurrence of diabetes mellitus. Higher BMI is a well-known risk factor for both hypertension and diabetes; however, the HIV-associated factors such as the CD4 count and the duration and type of ART regimen^36^ were not associated with either of the two conditions.

## Strengths and limitations

### Strengths

Our study was conducted in an SSA country, representing the highest HIV burden globally, with most (90.2%)^20^ of PLWH enrolled on ART. Our study is the first to report on the risk factors associated with CMD among PLWH, contributing to the knowledge gap identified in the SSA region.

Our findings can be used as a baseline for future planning for resource allocation for comprehensive management of HIV and the prediction of the likely economic implications for HIV care. The findings from this study can be used to assess the need for integration of HIV and NCD programmes. Other SSA countries of similar demographic characteristics and socio-economic status that still lack data on the burden and risk factors of CMDs and the associated risk factors can adapt the findings for baseline planning in their settings.

### Limitations

Although we took a random sample, there were very few participants enrolled on the PI-based regimen. The findings on the PI-based group cannot be generalised to the general population receiving PI-based regimens for HIV infection management.

Using secondary data is often associated with misclassification bias because of inappropriate recording of patients’ medical records; some participants shared similar unique numbers and hence, their documents were easily inappropriately filed. Finally, this study lacked ART-naïve PLWH or an uninfected age- and gender-matched group as a comparison to observe whether the incidence rates and risk factors were different between the two groups.

## Conclusion and recommendations

### Conclusion

The incidence of CMD in this patient population is low, yet noteworthy. There is a need for routine screening for CMDs in PLWH.

### Recommendations

To enable early detection and early stage treatment, we recommend that routine screening for CMDs should be included as part of the standard package among PLWH on ART in countries with a high burden of HIV, such as Lesotho. It has been confirmed that ART service delivery models aimed at decongesting local health facilities, encouraging patient retention in care and enhancing adherence to medication are more effective than corresponding facility-based care and superior to individual-based models.^37^ Integrating and harmonising HIV and NCD care services can greatly benefit the care of PLWH.
